# Case report: Hyperactive delirium after a single dose of zolpidem administered additionally to psychopharmacotherapy including clozapine

**DOI:** 10.3389/fpsyt.2023.1204009

**Published:** 2023-07-27

**Authors:** Maximilian Preiss, Ulrich Rabl, Valentin Popper, Victoria Watzal, Michael Treiber, Dominik Ivkic, Nicole Praschak-Rieder, Angela Naderi-Heiden, Gernot Fugger, Richard Frey, Dan Rujescu, Lucie Bartova

**Affiliations:** ^1^Department of Psychiatry and Psychotherapy, Clinical Division of General Psychiatry, Medical University of Vienna, Vienna, Austria; ^2^Comprehensive Center for Clinical Neurosciences and Mental Health, Medical University of Vienna, Vienna, Austria

**Keywords:** hyperactive delirium, anterograde amnesia, non-benzodiazepine, zolpidem, clozapine

## Abstract

The non-benzodiazepine hypnotic zolpidem is frequently administered as a short term psychopharmacotherapy for insomnia. Although it is well-established in a broad clinical routine and often well-tolerated, severe delirium and complex sleep behavior were reported in rare cases. Hereby, it remains unclear whether zolpidem's potential for delirium might be enhanced when combined with further psychopharmacotherapeutics. The present case report portrays a young male Caucasian inpatient with schizoaffective disorder, who was admitted due to severe hyperactive delirium after a single dose of zolpidem 10 mg that was administered in addition to already established psychopharmacotherapy including clozapine 200 mg/day, aripiprazole 15 mg/day and cariprazine 4.5 mg/day. In detail, disorientation, agitation, confabulations, bizarre behavior, and anterograde amnesia occurred shortly after ingestion of zolpidem and gained in intensity within a couple of hours. Once zolpidem was discontinued, the abovementioned symptoms subsided completely and did not reoccur. Since a clear temporal association could be drawn between the intake of zolpidem and the onset of hyperactive delirium, the present clinical experience should serve as a cautionary note for combining potent sedative-hypnotics and substances with anticholinergic properties, even in young adults in a good general condition. Moreover, our case argues for the necessity of further research into the pathomechanism of the interaction potential of non-benzodiazepines as zolpidem, especially with substances exerting anticholinergic properties, which are known for their potential to precipitate delirium. Therefore, the metabolic pathways of the concurrently administered substances should be further taken into account.

## 1. Introduction

Zolpidem is a gamma-aminobutyric acid (GABA_A_) receptor agonist of the imidazopyridine class that is primarily indicated for a short term (≤ 4 weeks) psychopharmacotherapy of insomnia (1). It acts as rapid and short-acting potent sedative with only minor anxiolytic, anticonvulsant, and muscle-relaxant properties (2). As agonist at the benzodiazepine receptor component of the GABA alpha-receptor complex, zolpidem mediates an inhibition on excitatory neurons. It is predominantly eliminated via the hepatic route into three pharmacologically inactive metabolites, mainly through the cytochrome P450 isoenzyme CYP3A4 (1, 2).

In patients with insomnia, efficacy of this non-benzodiazepine hypnotic has been shown to be comparable with benzodiazepines, while being less addictive. Although nausea, dizziness, drowsiness, and diarrhea have been described as common adverse effects (AEs) (1–3), zolpidem is commonly well-tolerated and largely accepted by the patients. Despite its broad employment in the clinical routine, severe AEs like anterograde amnesia, delirium, and complex sleep behavior have been observed occasionally and in some cases have led to grave incidents in patients and those around them (3–12). Whether zolpidem's potential for delirium might be triggered when combined with further psychopharmacotherapeutic agents has, however, not been systematically investigated yet. This might be of clinical relevance especially in case of combination treatments with agents exerting anticholinergic effects, which are known for their potential to precipitate delirium.

Here, we report a case of a young male Caucasian inpatient, who was admitted due to severe hyperactive delirium with disorientation, agitation, confabulations, bizarre behavior, and anterograde amnesia after a single dose of zolpidem 10 mg.

## 2. Case description

On the 20^th^ September 2022, a 34-year-old male patient of Caucasian ethnicity with a history of schizoaffective disorder of predominantly mixed type (ICD-10: F25.2, DSM-5: 295.70) was admitted to a general psychiatric ward of the Medical University of Vienna, Austria due to acute disorientation, agitation, and bizarre behavior. According to his spouse and parents, the patient had woken up around midnight of the day of admission and presented in a state of acute confusion and agitation. Contrary to a normal episode of sleep inertia, the aforementioned symptoms continued to intensify. Reportedly, the patient was partially oriented and not able to fall asleep again due to his state of agitation. He claimed to suffer from “a storm of thoughts” and elicited bizarre behavior with out of context comments and actions (e.g., denying the presence of his spouse beside him in bed despite her actual presence as well as consistently interpreting idiomatic expressions in a literal sense). Because his symptoms did not mitigate until midday, he was admitted to the psychiatric ward, where he had been receiving treatment in the course of the known schizoaffective disorder before. His daily medication upon admission comprised clozapine 200 mg, cariprazine 4.5 mg, aripiprazole 15 mg, and doxycycline 300 mg (cariprazine had been additionally prescribed by his outpatient psychiatrist for the past 2 weeks due to insufficient clinical improvement). On the ward the patient continued to present perplexed, only partially oriented in a fluctuating manner (e.g., believing it was winter and later realizing it was fall, or asserting that they had visited a winter's market before admission), and with vivid confabulations (e.g., claiming to have been fed oranges in front of the clinic and subjectively recalling arriving with a large bag instead of a small backpack, persistently searching for the abovementioned bag). He was not physically aggressive in any way toward others or himself. However, due to the hyperactive delirium and the state of agitation a constant 1:1 supervision by medical staff was necessary.

Regarding diagnostics, the patient's vital signs, urinary drug test, blood alcohol levels, and electrocardiography (ECG) were unremarkable. A comprehensive blood analysis showed normal results, with the exception of slightly elevated levels of alkaline phosphatase (139 U/L, RI: 40–130 U/L) and alanine transaminase (78 U/L, RI: <50 U/L). Plasma levels of clozapine were within therapeutic range, while aripiprazole showed values slightly below. In addition, The pharmacogenetic cytochrome P450 testing to determine drug-metabolizing capacity of the liver enzymes revealed an ultra-rapid metabolizer genotype for isoenzyme 1A2, with other findings of negligible importance. Moreover, during the last admission in August 2022 cranial magnetic resonance imaging (MRI) yielded no pathological findings, while a cerebrospinal fluid (CSF) analysis had shown elevated liquor/serum levels of borrelia antibodies. Tests for CSF pleocytosis, CXCL13-protein, borrelia-PCR, and borrelia culture turned out to be negative. The absence of neuroborreliosis-specific clinical symptoms, such as erythema migrans or cranial nerve palsy, in addition to no reported tick bites within the last 2 years, led to the interpretation of this finding as either a false-positive result or suggestive of an asymptomatic past infection (13). Nonetheless, in accordance with recommendations from the university department of neurology and in line with current guidelines (14), the patient has received a prophylactic antibiotic treatment with doxycycline from the 2^nd^ Sept. to the 23^rd^ Sept. 2023 (21 days).

Regarding the patient's history, the schizoaffective disorder first manifested 1.5 years prior, characterized by hyperactivity and vivid delusions without hallucinations or disorientation. In previous admissions, he mainly exhibited a depressed mood and mild delusions, effectively managed upon his last discharge in August 2022. The patient had no history of substance use disorder nor further relevant comorbidities. Of notable importance was the positive family history, specifically the occurrence of suicide during a severe depressive episode in the patient's older brother in 2007. Concerning his psychopharmacotherapy, the patient had previously taken zolpidem 10 mg on an as-needed basis, which was well-tolerated at the time. Subsequently, zolpidem was discontinued prior to the initiation of clozapine and aripiprazole. Moreover, the patient experienced a previous brief episode with disorientation and audiovisual hallucinations under clozapine 200 mg/day and bupropion 150 mg/day, which resolved quickly after discontinuing both. Clozapine was later incrementally and successfully reestablished due to its effectiveness in treating the patient's schizoaffective disorder.

**Figure 1:**
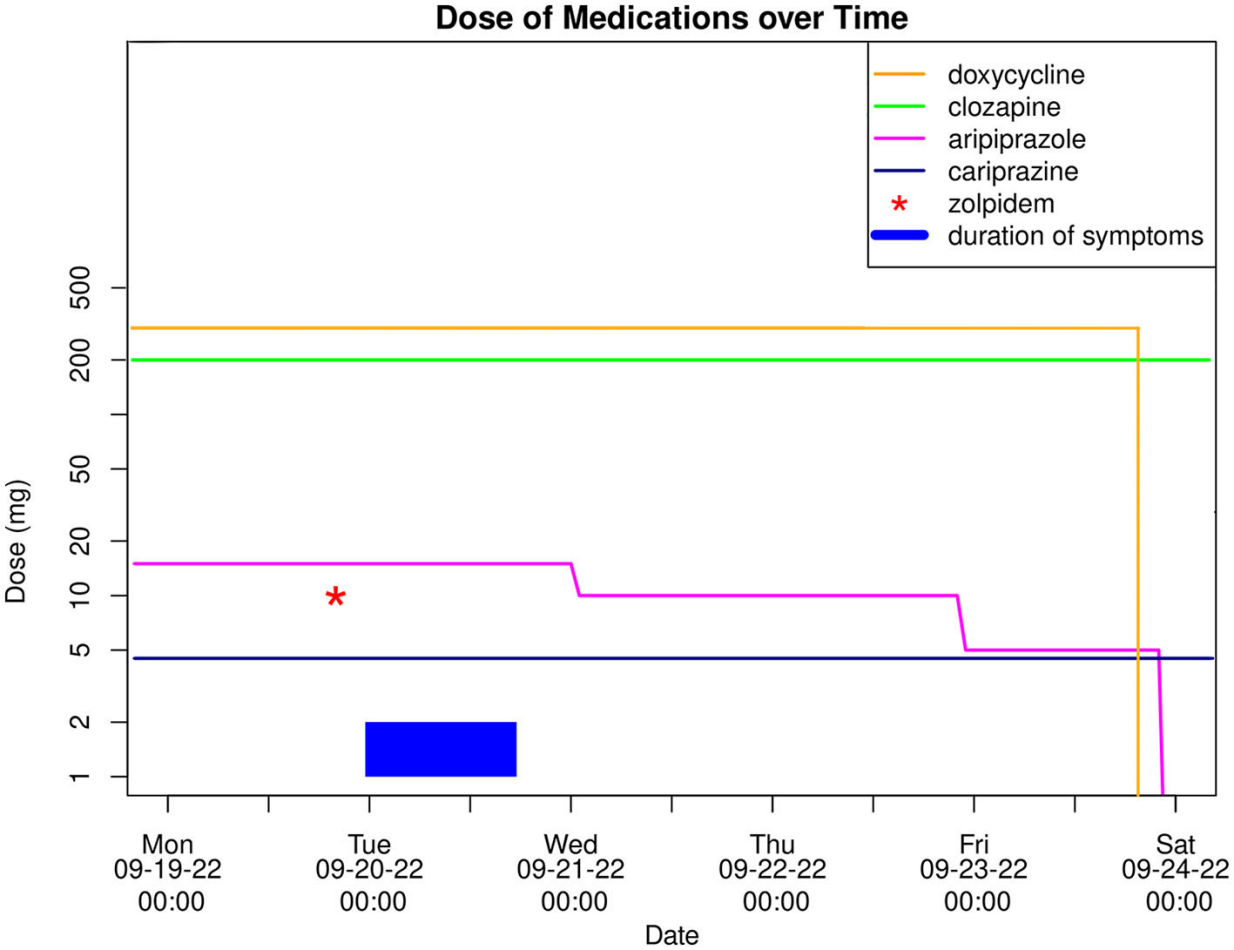
Graph showcasing a timeline of the case with dosage of medication (logarithmic scale) and duration of symptoms.

Toward the evening of admission, the symptoms finally subsided, and 17 h after their initial onset the patient was fully oriented again and did not experience any residual symptoms. Remarkably, suffering from anterograde amnesia he claimed to have fallen asleep in his bed at home and woken up in hospital. According to the patient's report, he had taken an single dose of zolpidem 10 mg ~4 h before the incident because of sleep disturbances without prior consultation of his outpatient psychiatrist. In the course of his inpatient treatment optimization, he was asked to avoid taking zolpidem in the future, while his ongoing antipsychotic combination treatment with clozapine 200 mg/day and cariprazine 4.5 mg/day was continued. Due to similar pharmacological properties of cariprazine and aripiprazole, which may potentiate AEs when combined (15), the latter was gradually discontinued (see Figure 1). During the following observation period of 6 days, none of the aforementioned symptoms reoccurred and the patient could be discharged from the hospital in a fully remitted state.

Upon follow-up approximately 8 months later, the patient reported to have remained stable on the unchanged medication without experiencing any further delirious symptoms. He attributed the delirious episode to zolpidem and reported abstaining from its use since then.

## 3. Discussion

The present case illustrates the occurrence of hyperactive delirium with anterograde amnesia in a young adult Caucasian male after a single dose of zolpidem 10 mg that was taken additionally to already established antipsychotic as well as an antibiotic treatment. The presumed causal association with zolpidem is supported by the fact that symptoms of delirium initiated shortly after ingestion of zolpidem, terminated after an approximate equivalent of five half-life circles of the drug (2), and did not reoccur once zolpidem was discontinued. According to the Naranjo Scale, which is an algorithm used to determine the likelihood of a specific drug causing an adverse clinical event, this case of hyperactive delirium can be classified as a probable adverse drug reaction to zolpidem (16). Moreover, this case was systematically documented and thoroughly discussed during one of our national psychopharmacotherapeutic conferences conducted by ÖAMSP (Austrian Institute for Drug Safety in Psychiatry), where medication-related adverse events are regularly evaluated in accordance with established protocols.

In this context it is noteworthy that in <1% of available patient cases, zolpidem has shown to elicit complex sleep behavior or delirium (17), sometimes resulting in significant self-harm or harm to others (5–7, 12). This non-benzodiazepine substance selectively binds to the benzodiazepine omega1-receptor subtype in the central nervous system, specifically to the alpha-1 subunit. The omega1-receptor subtype is implicated in memory loss (18), contributing to anterograde amnesia, while binding at the alpha-1 subunit seems essential for the hypnotic/sedative effects and other adverse events of zolpidem (19). These hypnotic AEs and delirium, according to literature, are dose-dependent and more common in older individuals (5, 6).

Nevertheless, zolpidem was taken only once at a standard dose and previously had been tolerated well, indicating that it could not be solely responsible in eliciting delirium in our patient. The involvement of doxycycline was ruled out, as there is currently no evidence suggesting its association with delirium, either independently or in interaction with zolpidem or antipsychotics (20). In rare cases of acute neuroborreliosis delirium was sometimes observed, typically accompanied by inflammatory signs (e.g., CSF pleocytosis) and neurological symptoms including paresis (21). Yet our patient did neither exhibit neurological symptoms nor signs of CSF pleocytosis and received ongoing antibiotic treatment, rendering the involvement of neuroborreliosis highly unlikely.

Clozapine, however, with its anticholinergic properties is known for the potential to elicit delirium (22). It can be posited that the combination of zolpidem with clozapine might have been a leading factor in the development of delirium, especially considering the prior occurrence of delirium induced by clozapine in this patient. Additionally, zolpidem, cariprazine, aripiprazole, and to some extent clozapine undergo hepatic metabolism via CYP3A4 (23). Competitive inhibition of CYP3A4 by these drugs could have raised zolpidem plasma levels, potentially playing a role in the onset of delirium. Furthermore, we are aware that the lack of titration of zolpidem from 5 to 10 mg may have exacerbated the condition. Lastly, the observed slightly elevated alanine transaminase levels might have pointed to already mildly impaired liver functioning, in turn further increasing zolpidem plasma levels and therefore its hypnotic effects.

Another potential contributing factor to the observed AEs could be explained by the D2 receptor competition associated with the antipsychotics aripiprazole, cariprazine (both characterized by long elimination half-lives), and clozapine. However, given that aripiprazole did not reach therapeutic plasma concentrations and was gradually discontinued only after the complete resolution of all AEs, the impact of D2 receptor competition in the development of delirium in this patient may be considered relatively minor.

## 4. Conclusion

Our experience with a patient exhibiting severe hyperactive delirium with anterograde amnesia after a single standard-dose of zolpidem in addition to already established psychopharmacotherapy including clozapine, arpipiprazole and cariprazine serves as a cautionary note for combining potent sedative-hypnotics with other psychopharmacotherapeutics sharing the same metabolizing pathways, even when treating young adults who are in good general condition. While it's important to acknowledge the limitations of generalizing from a single case report, the significance of this particular incident should not be overlooked, as it resulted in our patient's inadvertent hospitalization. Average daily doses of zolpidem have been reported to result in grave consequences for several patients and those around them before (4–6, 12). Although AEs of zolpidem are usually time-limited and fully reversible, they may bear a relevant hazard potential. Hence, further research should be conducted into the pathomechanism of the interaction potential of non-benzodiazepines like zolpidem, especially with substances exerting anticholinergic properties, which are known for their potential to provoke delirium. Furthermore, the present case report provides a valuable argument for routine laboratory evaluation including genetic testing of cytochrome P450 enzyme activity, which would inform in advance about the individual metabolizing status and therefore possible vulnerability to drug-related AEs.

## Data availability statement

The original contributions presented in the study are included in the article/supplementary material, further inquiries can be directed to the corresponding author.

## Ethics statement

Ethical review and approval was not required for the study on human participants in accordance with the local legislation and institutional requirements. The patients/participants provided their written informed consent to participate in this study. Written informed consent was obtained from the individual(s) for the publication of any potentially identifiable images or data included in this article.

## Author contributions

MP wrote the case report including the first draft of the manuscript that was further elaborated and critically revised by LB. All listed authors were meaningfully involved in the performance of the reported therapy and the treatment of the patient and managed the literature search and have reviewed and approved the final manuscript.
